# Capgermacrenes A and B, Bioactive Secondary Metabolites from a Bornean Soft Coral, *Capnella* sp.

**DOI:** 10.3390/md13053103

**Published:** 2015-05-19

**Authors:** Chin-Soon Phan, Shean-Yeaw Ng, Eun-A Kim, You-Jin Jeon, Kishneth Palaniveloo, Charles Santhanaraju Vairappan

**Affiliations:** 1 Laboratory of Natural Products Chemistry, Institute for Tropical Biology and Conservation, Universiti Malaysia Sabah, 88400 Kota Kinabalu, Sabah, Malaysia; E-Mails: yuna123@hotmail.my (C.-S.P.); sheanyeaw90@hotmail.com (S.-Y.N.); kishneth@yahoo.com (K.P.); 2 Department of Marine Life Sciences, Jeju National University, Jeju 690-756, Korea; E-Mail: yellow6798@hanmail.net

**Keywords:** soft coral, *Capnella* sp., *Nephtheidae*, sesquiterpenoids, bicyclogermacrene

## Abstract

Two new bicyclogermacrenes, capgermacrenes A (**1**) and B (**2**), were isolated with two known compounds, palustrol (**3**) and litseagermacrane (**4**), from a population of Bornean soft coral *Capnella* sp. The structures of these metabolites were elucidated based on spectroscopic data. Compound **1** was found to inhibit the accumulation of the LPS-induced pro-inflammatory IL-1β and NO production by down-regulating the expression of iNOS protein in RAW 264.7 macrophages.

## 1. Introduction

**Figure 1:**
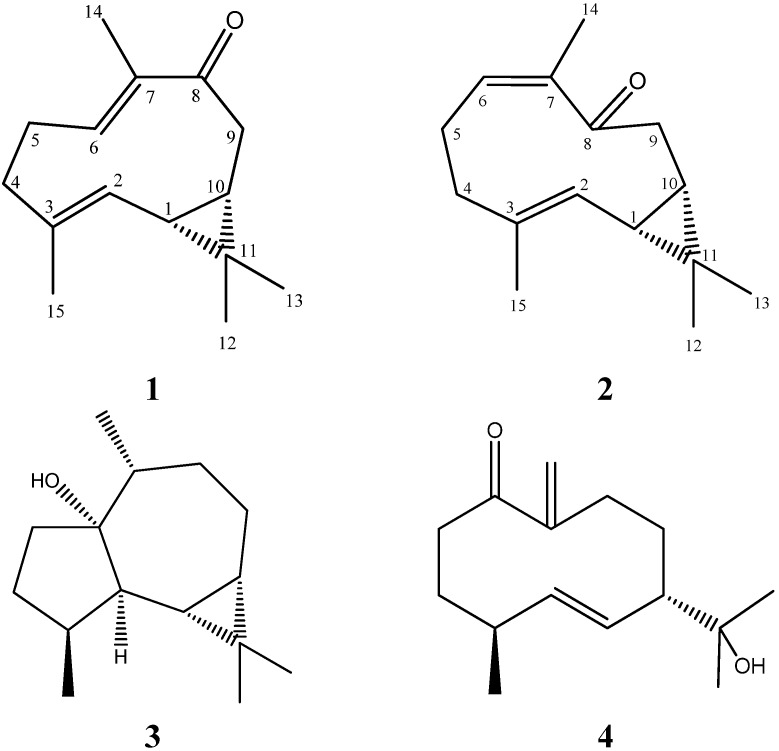
Structures of metabolites **1**–**4**.

Soft corals belonging to the genus *Capnella* (*Alcyonacea*, *Nephtheidae*) are known to produce sesquiterpenoids, such as capnellenes and capnellanes [1]. Soft coral derived secondary metabolites are reported to exhibit promising biological activities such as anti-tumor, antiviral, antifouling and anti-inflammation [2]. Our previous chemical investigation on the soft coral genus *Nephthea* led to isolation and structural elucidation of a new cembrane diterpene [3], a new sterol [4] and a new norsesquiterpenoid [5], along with several known compounds [5]. However, there were no sesquiterpenes isolated from Bornean soft coral genus *Capnella*. Therefore, we investigated a population of *Capnella* sp. from Mantanani Island (Sabah, Malaysia) and it has led to the isolation of bicyclogermacrenes as first report in Bornean soft corals. The methanol extract gave two new bicyclogermacrenes, capgermacrenes A (**1**) and B (**2**), in addition to two known compounds, palustrol (**3**) [6] and litseagermacrane (**4**) [7,8] (Figure 1). Anti-inflammatory activity of capgermacrene A (**1**) in LPS-induced RAW 264.7 macrophages exhibited down-regulation of inflammatory mediator such as nitric oxide (NO), prostaglandin-E2 (PGE_2_), tumor necrosis factor-α (TNF-α), interleukin 1β (IL-1β), interleukin 6 (IL-6), inducible nitric oxide synthase (iNOS) and cyclooxygenase-2 (COX-2). This paper reports the isolation, structure elucidation and anti-inflammatory activity of these four compounds.

## 2. Results and Discussion

### 2.1. Structure Determination

Compound **1** was isolated as colorless oil. High-resolution mass spectrometry data gave a molecular formula of C_15_H_22_O (*m*/*z* 219.1720 [M + H]^+^). The IR spectrum suggested the presence of α,β-unsaturated carbonyl group (1662 cm^−1^) in the molecule. The ^13^C-NMR spectral data of **1** (Table 1) revealed the presence of 15 carbon atoms, including four methyls, three methylenes, four methines and four quaternary carbons, based on DEPT-135 and HSQC spectra, suggesting a chemical skeleton of sesquiterpenoid. The NMR signals of **1** (Table 1) showed the presence of a ketone group at δC 206.6 (C), two trisubstituted double bonds at δ_C_ 146.5 (CH), 137.1 (C), 135.1 (C) and 125.2 (CH) (δH 6.09 (1H, ddq, *J* = 12.4, 3.4, 1.4 Hz) and 4.87 (1H, d, *J* = 10.3 Hz)), a cyclopropyl moiety at δC 26.1 (CH), 29.6 (CH) and 20.2 (C) (δH 1.32 (1H, dd, *J* = 10.3, 8.9 Hz) and 0.77 (1H, ddd, *J* = 11.7, 8.9, 2.8 Hz)), and four tertiary methyls at δC 13.3 (CH_3_), 16.7 (CH_3_), 15.9 (CH_3_) and 29.3 (CH_3_) (δH 1.74, 1.49, 1.12 and 1.08 (each 3H, s)) (see ^1^H- and ^13^C-NMR data of **1** in Supplementary Information). Five degrees of unsaturation calculated *via* HR-MS could be attributed to three double bonds and a bicyclic sesquiterpenoid framework leading to the structure of compound **1**.

**Table 1:** ^1^H- and ^13^C-NMR data (600 MHz and 150 MHz, in CDCl_3_) of **1** and **2** (δ in ppm, *J* in Hz).

Position	1		2	
	δC	δH	δC	δH
1β	26.1 (CH)	1.32 dd (10.3, 8.9)	27.9 (CH)	1.45 t (8.9)
2	125.2 (CH)	4.87 d (10.3)	122.9 (CH)	4.61 d (8.9)
3	137.1 (C)		137.6 (C)	
4α	38.9 (CH_2_)	2.34 m	39.3 (CH_2_)	1.58 t (11.7)
β				2.15 dd (11.7, 8.3)
5α	25.1 (CH_2_)	2.47 td (12.4, 8.3)	23.7 (CH_2_)	2.25 td (12.2, 8.3)
β		2.31 m		2.01 dt (12.2, 8.3)
6	146.5 (CH)	6.09 ddq (12.4, 3.4, 1.4)	130.2 (CH)	5.40 tq (8.3, 1.4)
7	135.1 (C)		140.0 (C)	
8	206.6 (C)		211.5 (C)	
9α	37.1 (CH_2_)	2.77 t (11.7)	36.3 (CH_2_)	2.32 d (8.9)
β		2.37 dd (11.7, 2.8)		
10β	29.6 (CH)	0.77 ddd (11.7, 8.9, 2.8)	28.3 (CH)	1.38 q (8.9)
11	20.2 (C)		21.7 (C)	
12α	15.9 (CH_3_)	1.12 s	16.1 (CH_3_)	1.11 s
13β	29.3 (CH_3_)	1.08 s	29.2 (CH_3_)	1.11 s
14	13.3 (CH_3_)	1.74 s	21.9 (CH_3_)	1.88 s
15	16.7 (CH_3_)	1.49 s	17.4 (CH_3_)	1.55 s

**Figure 2:**
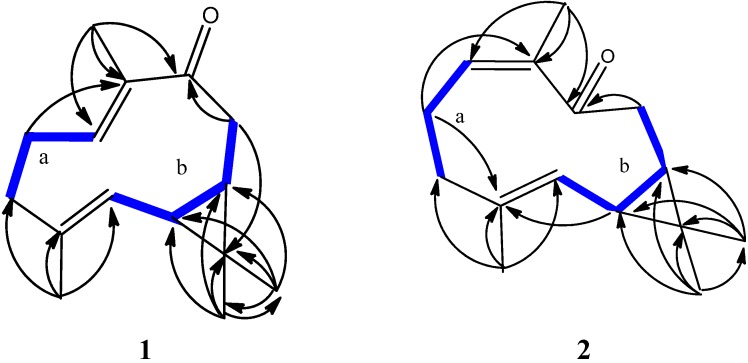
Selected ^1^H-^1^H COSY (▬) and HMBC (→) correlations of **1** and **2**.

Assignment of ^1^H-^13^C correlations of **1** was determined by HSQC analysis. Two separate consecutive spin systems of “a” and “b” of **1** were revealed by ^1^H-^1^H COSY correlations. Correlations corresponding to H_2_-4/H_2_-5/H-6 are represented by the “a” spin system and H-1/H-2/H_2_-9/H-10 by the “b” spin system, and are depicted by the bold lines in Figure 2. Both the “a” and “b” structural units were assembled to establish a bicyclic system comprised of fused 10- and 3-membered rings, suggesting a bicyclogermacrene skeleton for **1**. This was achieved using key HMBC correlations of H_3_-12α to C-1, C-10, C-11 and C-13; H_3_-13β to C-1, C-10, C-11 and C-12; H_3_-14 to C-6, C-7 and C-8; and H_3_-15 to C-2, C-3 and C-4 as depicted by arrows in Figure 2. Thus, **1** was deduced to possess a 10-membered ring with a ketone group at C-8, and a cyclopropyl moiety located at the C-1/C-10. This cyclopropyl ring was confirmed by the “b” partial structure based on the presence of HMBC correlations between H_3_-12 to C-1, C-10, C-11 and C-13; and H_3_-13 to C-1, C-10, C-11 and C-12. In addition, the upfield chemical shifts of H-6β (δ 1.32) and H-7β (δ 0.77), further supported the presence of this cyclopropyl moiety [9]. Based on these analyses, the planar structure of **1** was determined to be as shown in Figure 2.

**Figure 3:**
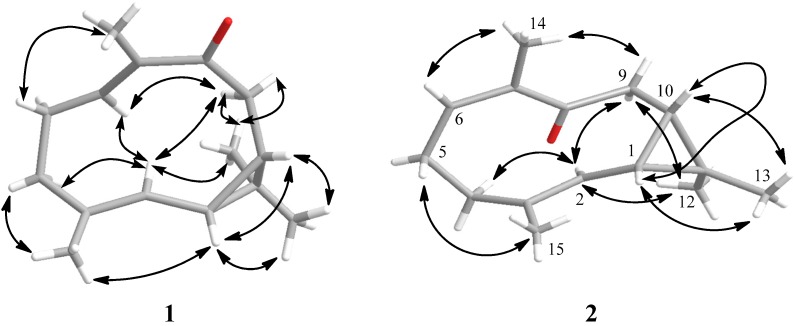
Selective correlations of NOESY for **1** and **2**.

The relative stereochemistry of compound **1** was deduced from NOESY correlations as shown in Figure 3. In addition, the double bonds geometries were deduced from the ^13^C-NMR chemical shifts at C-14 (δC 13.3) and C-15 (δC 16.7), which suggested *E*-configurations [10,11]. Besides, the lack of NOE correlations between H-6 with H_3_-14 and H-2 with H_3_-15, further supported this configuration. Other NOE analyses, H-6 showed NOE correlations to H-2, H_2_-5α and H_2_-9α; and H-2 showed NOE correlations with H_2_-4α, H-6, H_2_-9α and H_3_-12α. Therefore, H_2_-4α, H_2_-5α, H_2_-9α, and H_3_-12α are suggested to be of the similar orientation, reflecting an α-orientation, hence H_2_-4β, H_2_-5β and H_2_-9β are suggested to be of β-orientation. Furthermore, H-1β, H-10β, and H_3_-13β were confirmed to be positioned on the β-orientation, due to NOE correlations observed between H-1β with H-10β, H_3_-13β, and H_3_-15. Besides that, the scalar coupling value between H-1β and H-10β was 8.9 Hz and this coupling value suggested a *cis*-cyclopropyl group with both methine protons as β-orientated [12]. Based on these configurations, the relative structure of **1** is reported as (1*S*,10*R*,2*E*,6*E*)-3,7,11,11-tetramethylbicyclo(8.1.0)undeca-2,6-dien-8-one. The compounds **1** and **2** were *trans* and *cis* isomers, but downfield shifts were observed at H-1β in **1** and **2**, and H-10β in **2**. The downfield shifts of both cyclopropane methines for **2** (δH 1.45 and 1.38) were similar to those observed in cyclocolorenone (δH 1.54 and 1.29) and its derivative [13,14]. It is reported that this cyclocolorenone also has one double bond beside these cyclopropane methines similar to those of **1** and **2**, where the double bond was located at C2/C3. Thus, it could be due to the presence of this double bond at C2/C3 and its proton deshielding ability towards cyclopropane methines in **1** and **2**. However, the H-10β (δH 0.77) in **1** was relatively more shielded as compared to those of **2**. This could be due to the presence of *trans* geometry double bond at C6/C7 and the position of oxygen atom (carbonyl group was oriented upward and outside the 10-membered ring) of **1** as compared that of **2**. The chemical shifts of H-6 and C-6 in **1** were significantly downfield compared to those observed for **2**, possibly due to the mesomeric effect [15].

Compound **2** was obtained as colorless crystals. Its molecular formula, C_15_H_22_O was established by HR-MS (*m*/*z* 219.1725 [M + H]^+^), revealing five degrees of unsaturation. The IR spectrum indicated the presence of α,β-unsaturated carbonyl functionality (1683 cm^−1^) in the molecule. Comparison of the NMR data (Table 1) of **2** with those of **1** also revealed the structure **2** to be of bicyclogermacrene type. Based on 2D NMR spectra, two separate consecutive proton spin systems “a” and “b” of **2** were revealed in ^1^H-^1^H COSY spectrum (Figure 2); “a” spin system for H_2_-4/H_2_-5/H-6 and “b” for H-1/H-2/H_2_-9/H-10. Planar structure of **2** was established based on HMBC correlations. The relative configurations of the two successive chiral centers at C-1 and C-10 in **2** were determined based on NOESY data, as shown in Figure 3. It was revealed that H-6 had NOE correlation with H_3_-14 and H-2 had no NOE correlation with H_3_-15, implying the *Z-* and *E*-configurations for double bonds at C-6/C-7 and C-2/C-3, respectively. Therefore, by comparison of the ^13^C-NMR of C-14 of **2** with that of **1**, it was suggested that **2** had a *cis* and **1** had a *trans* double bond at C-6/C-7 [10,11]. In addition, H-2 showed NOE interactions with H_2_-4α, H_2_-9α, and H_3_-12α; and H_3_-15 exhibited NOE correlation with H_2_-5α. Therefore, H_2_-4α, H_2_-5α, H_2_-9α, and H_3_-12α reflected an α-orientation, hence indicating H_2_-4β, H_2_-5β and H_2_-9β were β-orientated. Further analysis of other NOE correlations and scalar coupling between H-1β and H-10β, revealed **2** possessed the similar relative configurations at C-1, C-10, and C-13 as that of **1**. On the basis of above findings, the relative structure of **2** is reported as (1*S*,10*R*,2*E*,6*Z*)-3,7,11,11-tetramethylbicyclo(8.1.0)undeca-2,6-dien-8-one. The ^1^H- and ^13^C-NMR chemical shift at C-6 of **2** were more shielded as compared to those of **1** due to the absence of mesomeric effect.

### 2.2. Anti-Inflammatory Properties

**Figure 4:**
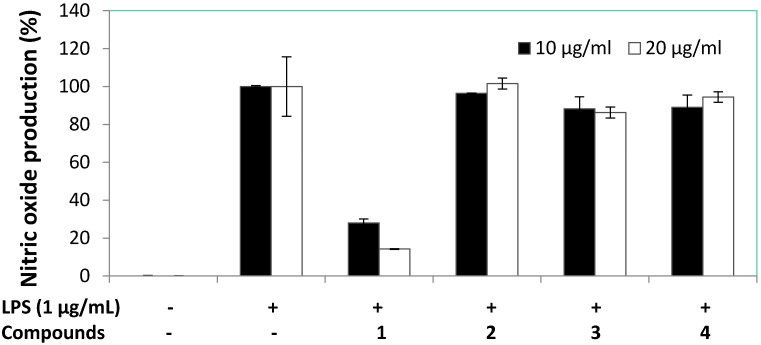
The effect of compounds **1**–**4** on NO production in RAW 264.7 macrophages.

**Figure 5:**
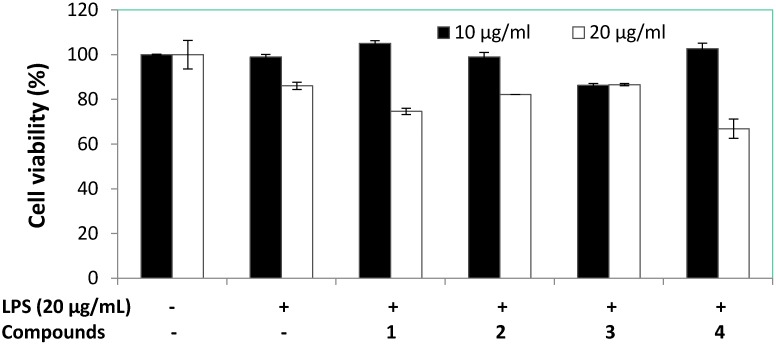
The effect of compounds **1**–**4** on cell viability in RAW 264.7 macrophages.

**Figure 6:**
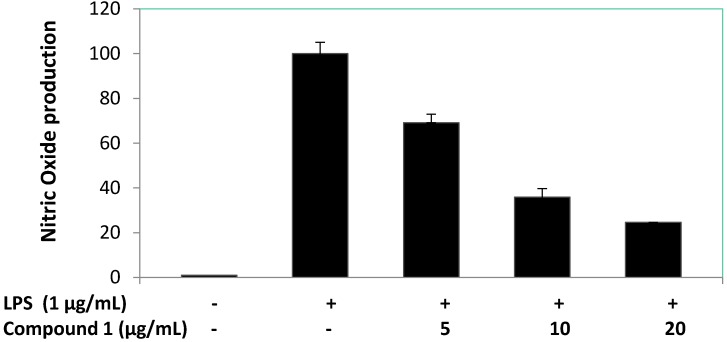
Concentration dependent effects of compound **1** on NO production in RAW 264.7 macrophages.

Anti-inflammatory activity bioassay of compounds **1**–**4** against RAW 264.7 macrophages were evaluated based on the accumulation of NO production and cell viability induced by LPS (1 μg/mL) (Figure 4 and Figure 5). The results showed compound **1** displayed potent anti-inflammatory potential by significantly reducing the NO production of LPS-induced RAW macrophages to 28.0% and 14.2% at 10 and 20 μg/mL, respectively. Cell viability of LPS-induced RAW macrophages in the presence of compound **1** was 105.1% and 74.6% at 10 and 20 μg/mL, respectively. Therefore, compound **1** was selected for further anti-inflammatory investigation against accumulation of NO, PGE_2_, and pro-inflammatory cytokines (TNF-α, IL-1β, and IL-6) production and the expression of iNOS and COX-2 proteins induced by LPS (1 μg/mL) in RAW 264.7 cells were evaluated. The effect of compound **1** on NO production in LPS-treated RAW 264.7 macrophages was repeated at lower concentrations, as shown in Figure 6. The findings showed NO production of compound **1** was 69.1%, 60.0%, and 24.6% at the concentrations of 5, 10, and 20 μg/mL, respectively. Based on the cell viability screening, compound **1** revealed the potential to significantly inhibit LPS-induced NO production in a concentration dependent manner, also suggesting absence of cytotoxic effects in RAW 264.7 macrophage cells. Dexamethasone used as positive control inhibited NO production to 16.01% at 5.0 μg/mL. At the concentration of 12.51 ± 0.16 μg/mL (57.34 μM) of compound **1**, it inhibited 50% of NO production in LPS-stimulated RAW 264.7 macrophages.

**Figure 7:**
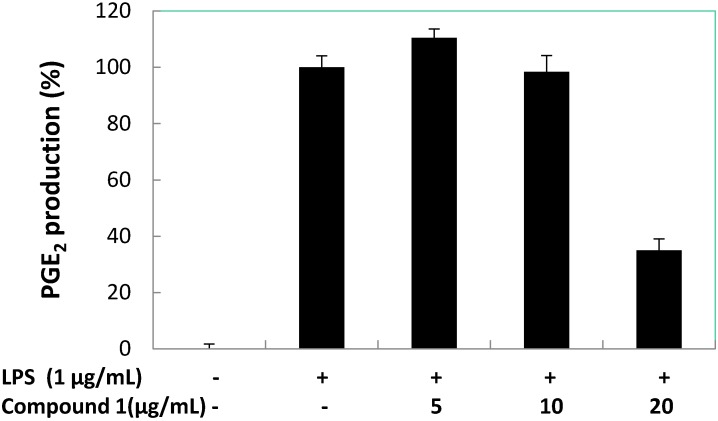
Concentration dependent effects of compound **1** on LPS-induced PGE_2_ production in RAW 264.7 macrophages.

Further, we evaluated the effect of compound **1** on the LPS-induced production of PGE_2_ in RAW 264.7 macrophage cells (Figure 7). However, it was apparent that compound **1** was not a good inhibitor of PGE_2_ production in LPS-treated RAW 264.7 macrophages at 5 and 10 μg/mL, but compound **1** reduced the PGE_2_ production to below 40% at 20 μg/mL. Dexamethasone (positive control) inhibited PGE_2_ production to 11.58% at 5.0 μg/mL. Compound **1** at 18.97 ± 0.63 μg/mL (86.93 μM) inhibited 50% of PGE_2_ production in LPS-stimulated RAW 264.7 macrophages.

**Figure 8:**
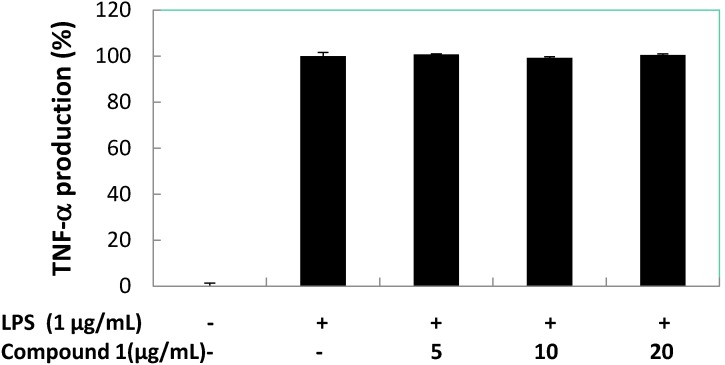
Concentration dependent effects of compound **1** on LPS-stimulated TNF-α release in RAW 264.7 cells.

**Figure 9:**
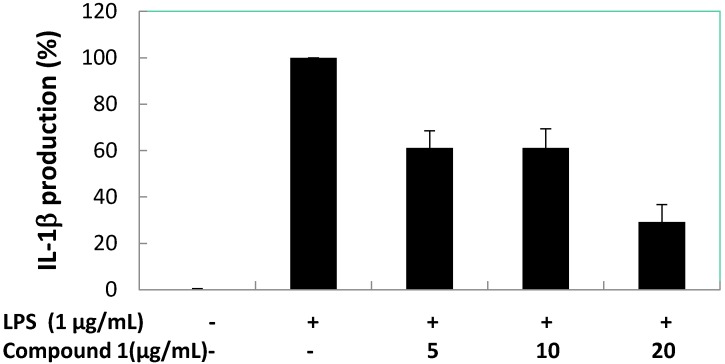
Concentration dependent effects of compound **1** on LPS-treated IL-1β release in RAW 264.7 macrophage cells.

**Figure 10:**
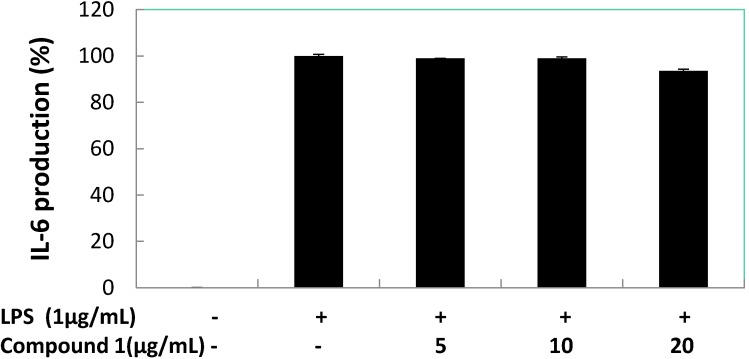
Concentration dependent effects of compound **1** on LPS-induced IL-6 release in RAW 264.7 macrophage cells.

Subsequently, the effects of compound **1** on pro-inflammatory cytokines (TNF-α, IL-1β, and IL-6) production induced by LPS in RAW 264.7 macrophage cells were quantified (Figure 8, Figure 9 and Figure 10). The result shows there was almost no inhibition of TNF-α and IL-6 production by compound **1** at 5, 10, and 20 μg/mL. However, compound **1** was able to inhibit the production of IL-1β with a reduction of 40% compared to the LPS-induced group at 5 and 10 μg/mL, and the IL-1β production value dropped to below 35% when 20 μg/mL of compound **1** was used. At 5.0 μg/mL of positive control (dexamethasone) inhibition of TNF-α, IL-1β and IL-6 reduced production to 15.31, 12.23 and 13.56%, respectively. At the concentration of 12.89 ± 1.38 μg/mL (59.06 μM) compound **1**, inhibited 50% of IL-1β production in LPS-stimulated RAW 264.7 macrophages.

**Figure 11:**
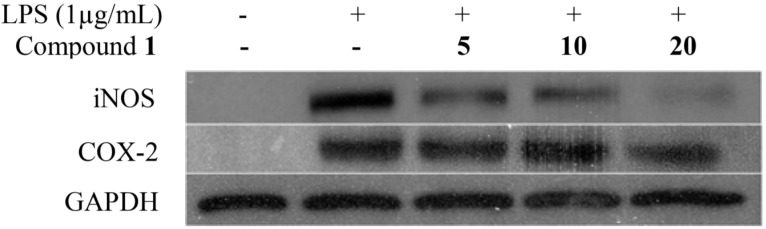
Concentration dependent effects of compound **1** on protein and mRNA expressions of iNOS and COX-2 in LPS-induced RAW 264.7 macrophages.

Further investigation pertaining to the mechanism of anti-inflammatory activity of compound **1** was evaluated by observing the expression of iNOS and COX-2 proteins via Western blot, as shown in Figure 11. The result showed pretreatment with compound **1** significantly inhibited iNOS protein expression in a concentration dependent manner. However, compound **1** showed little inhibition of COX-2 protein expression at first concentration at 5 μg/mL, followed by 10 and 20 μg/mL by visual observation of bands densities, indicating little inhibition of PGE_2_ production in LPS-treated RAW 264.7 macrophages. In this anti-inflammatory assay, we confirmed that LPS had significantly increased TNF-α, IL-1β, IL-6, PGE_2_ and NO production, which resulted in the over expression of iNOS and COX-2. Pretreatment of LPS-induced RAW 264.7 macrophages with compound **1** exhibited the ability to inhibit NO and IL-1β production by down-regulating expression of iNOS. In addition, compound **1** also showed little inhibition of PGE_2_ by little suppression of COX-2 expression.

Compound **1** and **2** were *trans* and *cis* isomers, but only compound **1** showed anti-inflammatory activity. It is our assumption that the differences in activity between these two compounds are due to their stereo differences. Compound **1** has a larger space of 10-membered ring as compared those of **2**. The oxygen atom of **2** was positioned inside of the 10-membered ring, but oxygen atom of **1** was orientated outside of the 10-membered ring. Thus, the anti-inflammatory activity of compound **1** could be attributed to the larger space within the 10-membered ring and the position of oxygen atom. However, further SAR (Structure Activity Relation) analysis would be required to confirm this phenomenon.

## 3. Experimental Section

### 3.1. General

^1^H-NMR (600 MHz) and ^13^C-NMR (150 MHz) spectra were recorded on a JEOL ECA 600 FT-NMR using CDCl_3_ with TMS as an internal standard. The high-resolution mass spectrum was acquired via LCMS-IT-TOF (Shimadzu, Nakagyo-ku, Kyoto 604-8511, Japan). AUTOPOL IV automatic polarimeter (Rudolph Research Analytic, 55 Newburgh Road, Hackettstown, USA) was used to measure the optical rotation. Infrared spectra were recorded on a fourier transform infrared spectroscopy (Thermo Nicolet, 81 Wyman Street, Waltham, MA, USA). Preparative TLC was performed with silica gel glass plates (Merck, Kieselgel 60 F_254_, Menara Sunway Annexe Jalan Lagoon Timur (PJS9/1), Bandar Sunway, Malaysia). Column chromatography was performed with silica gel (Merck, Kieselgel 60, 70–230 mesh). Precoated silica gel plates (Merck Kieselgel 60 F_254_) were used for analytical TLC. Both the UV light (254 and 365 nm) and spraying 5% phosphomolybdic acid-ethanol solution were used for spots visualization. High-performance liquid chromatography was performed on the Shimadzu prominence components using UV detector with a phenomenex C18 column (250 × 10 mm, 5 μm).

### 3.2. Biological Material

The specimen of *Capnella* sp. was collected from Mantanani Island, Sabah (6°43.059″N, 116°20.189″E), in June 2013. The voucher specimen (JUN117BOR) was deposited in the BORNEENSIS Collection of Institute for Tropical Biology and Conservation, Universiti Malaysia Sabah.

### 3.3. Extraction and Isolation

The fresh soft coral (1 kg wet wt) was chopped and extracted with MeOH at room temperature for 7 days. The resulting MeOH was concentrated under reduced pressure and the residue was partitioned between EtOAc and H_2_O. The EtOAc fraction was further partitioned with hexane and 90% MeOH. The 90% MeOH fraction (1.20 g) was subjected to column chromatography eluting with a gradient of hexane and EtOAc in an increasing polarity to obtain six fractions. During column chromatography separation step, fractions 1, 2 and 3 were obtained by elution with hexane-EtOAc (9:1), (8:2) and (7:3), respectively. A portion of fraction 2 (80.0 mg) was subjected to preparative TLC with toluene-EtOAc (95:5) to yield compound **1** (11.8 mg) and compound **2** (8.5 mg),and was purified by reverse-phase HPLC (gradient mode; MeCN-H_2_O (1:1) as solvent A, 100% MeCN as solvent B, 0.1–15.0 min with 50% of solvent B, 15.1–45.0 min from 50% to 100% of solvent B, and compound **2** peak retention time was at 25 min). A portion of fraction 1 (150.0 mg) was submitted to repeated preparative TLC with toluene, hexane–EtOAc (9:1) and toluene to afford compound **3** (6.0 mg). Fraction 3 (64.3 mg) was submitted to repeated preparative TLC with hexane–EtOAc (3:1) and CHCl_3_-EtOAc (9:1) to yield compound **4** (1.2 mg).

### 3.4. Capgermacrene A (**1**)

Colorless oil; [α]_D_^25^: +73.6 (*c* 0.47, CHCl_3_); IR (KBr) λ_max_ 1662cm^−1^; ^1^H-NMR and ^13^C-NMR spectral data: see Table 1; HR-TOFMS *m*/*z* 219.1720 [M + H]^+^ (calcd. for C_15_H_23_O, 219.1743), and *m*/*z* 437.3359 [2M + H]^+^ (calcd. for C_30_H_45_O_2_, 437.3407).

### 3.5. Capgermacrene B (**2**)

Colorless crystals; [α]_D_^25^: −21.7 (*c* 0.81, CHCl_3_); IR (KBr) λ_max_ 1683 cm^−1^; ^1^H-NMR and ^13^C-NMR spectral data: see Table 1; HR-TOFMS *m*/*z* 219.1725 [M + H]^+^ (calcd. for C_15_H_23_O, 219.1743).

### 3.6. In Vitro Anti-Inflammatory Assay

RAW 264.7 cells were purchased from the Korean Cell Line Bank (KCLB; Seoul, Korea). The enzyme-linked immunosorbent assay (ELISA) kit for PGE_2_, TNF-α, IL-1β, and IL-6 were obtained from R&D Systems Inc. (Minneapolis, MN, USA). The antibodies against iNOS and COX-2 were purchased from Calbiochem (La Jolla, CA, USA) and BD Biosciences Pharmingen (San Jose, CA, USA), respectively. The anti-inflammatory assay was from known procedure [16,17].

The cytotoxicity assay, determination of nitric oxide (NO), and pro-inflammatory cytokines (TNF-α, IL-1β and IL-6) productions, was carried out by cultured RAW 264.7 cell line and seeded in 96-well plate at concentration of 1.0 × 10^5^ cells mL^−1^. Upon incubation, the cells were treated with compound **1** or compounds **1**–**4** followed by LPS (1 μg/mL). In cytotoxicity assay, 3-(4,5-dimethylthiazole-2-yl)-2,5-diphenyltetrazolium bromide (MTT) stock solution was added in each well. Subsequently, the absorbance was measured using ELISA. In evaluation of NO production, griess reagent (1% sulfanilamide and 0.1% naphthylethylenediamine dihydrochloride in 2.5% phosphoric acid) was used. The optical density was measured using ELISA. Besides that, determination of pro-inflammatory cytokines was carried out using ELISA to report absorbance. Western blot analysis was carried out with compound **1** with a series of chemical reagents, incubation and centrifugation. Subsequently, the membrane was incubated with anti-mouse iNOS (1:1000; Calbiochem, San Diego, CA, USA) and anti-mouse COX-2 (1:1000; BD Biosciences Pharmingen, San Diego, CA, USA) overnight at room temperature. Then, the bands were visualized on X-ray film using ECL detection reagent (Amersham Biosciences, Piscataway, NJ, USA).

## 4. Conclusions

As part of our ongoing interest in chemical investigation of Bornean soft corals, two new bicyclogermacrenes, capgermacrene A (**1**) and B (**2**), were isolated together with two known compounds, palustrol (**3**) and litseagermacrane (**4**), from a *Capnella* sp. population collected from Mantanani Island, Sabah. This investigation has enriched our knowledge pertaining to the diversity of secondary metabolites in Bornean soft corals. Compound **1** and **2** could be regarded as relatively rare bicyclogermacrene structures isolated from *Capnella* sp. The anti-inflammatory activity and structure related activity of compound **1** revealed to be very interesting due to the mere differences in their stereochemistry. Upon detailed SAR and pharmacokinetic investigation, compound **1** could be a promising iNOS inhibiting agent.

## Supplementary Information

Structures of new compounds, 1D and 2D NMR spectra, and MS spectra of the new compounds (**1** and **2**).
