# Supplementary material for: Capgermacrenes A and B, Bioactive Secondary Metabolites from a Bornean Soft Coral, Capnella sp
Source: Mar Drugs. 2015 May 19;13(5):3103–15. doi: 10.3390/md13053103 (PMC4446621; doi:10.3390/md13053103)
Supplement: Supplementary file 1 [file marinedrugs-13-03103-s001.pdf]

Supplementary Information

Figure S1. ^{1}H-NMR spectrum of capgermacrene A (**1**) in $CDCl_3$ (600 MHz).
Figure S2. ^{13}C-NMR spectrum of capgermacrene A (**1**) in $CDCl_3$ (150 MHz).
Figure S3. DEPT-135 spectrum of capgermacrene A (**1**) in $CDCl_3$.
Figure S4. HSQC spectrum of capgermacrene A (**1**) in $CDCl_3$.
Figure S5. ^{1}H-^{1}H COSY spectrum of capgermacrene A (**1**) in $CDCl_3$.
Figure S6. HMBC spectrum of capgermacrene A (**1**) in $CDCl_3$.
Figure S7. NOESY spectrum of capgermacrene A (**1**) in $CDCl_3$.
Figure S8. HR-TOFMS data of capgermacrene A (**1**).
Figure S9. ^{1}H-NMR spectrum of capgermacrene B (**2**) in $CDCl_3$ (600 MHz).
Figure S10. ^{13}C-NMR spectrum of capgermacrene B (**2**) in $CDCl_3$ (150 MHz).
Figure S11. DEPT-135 spectrum of capgermacrene B (**2**) in $CDCl_3$.
Figure S12. HSQC spectrum of capgermacrene B (**2**) in $CDCl_3$.
Figure S13. ^{1}H-^{1}H COSY spectrum of capgermacrene B (**2**) in $CDCl_3$.
Figure S14. HMBC spectrum of capgermacrene B (**2**) in $CDCl_3$.
Figure S15. NOESY spectrum of capgermacrene B (**2**) in $CDCl_3$.
Figure S16. HR-TOFMS data of capgermacrene B (**2**).

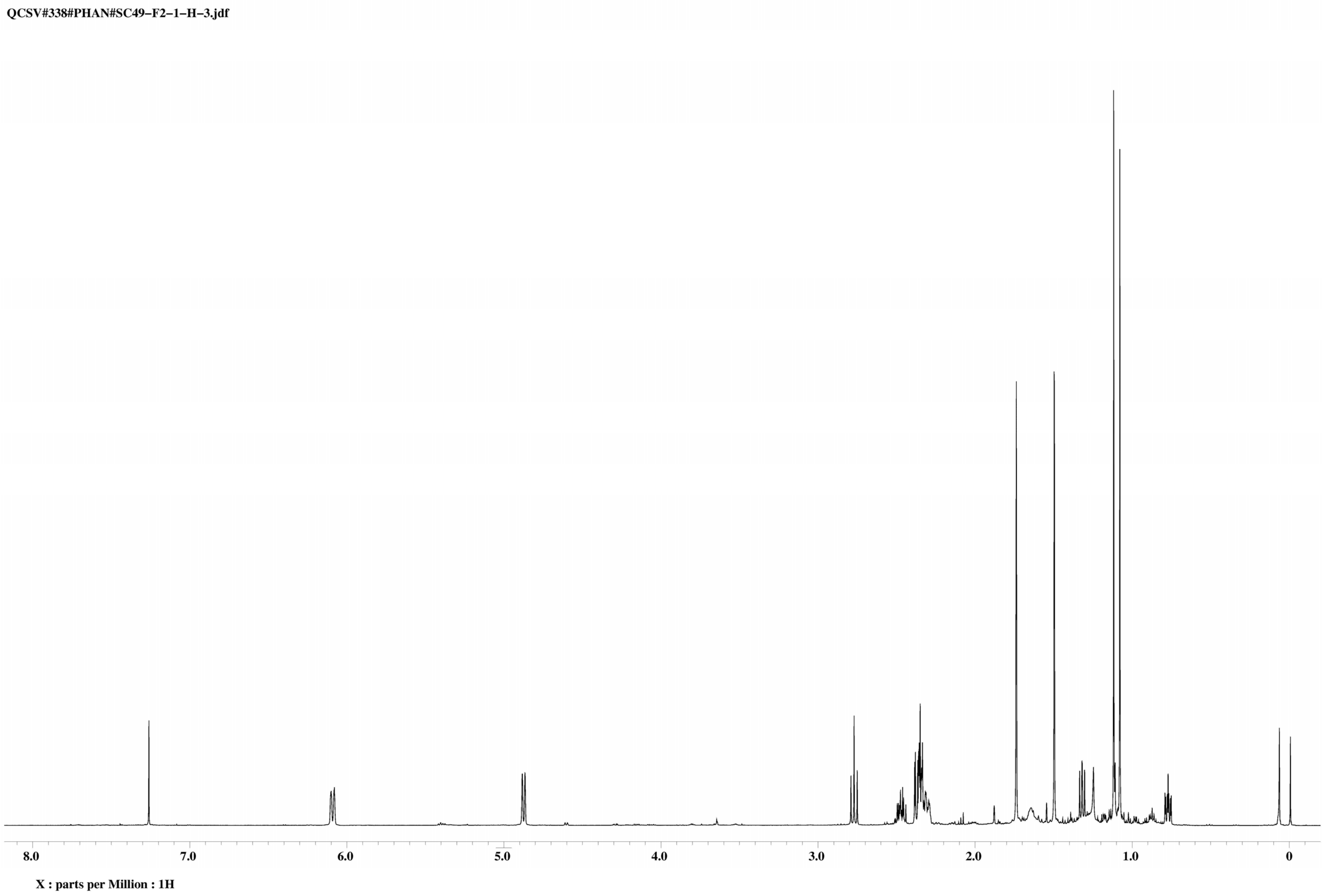

Figure S1. ^{1}H-NMR spectrum of capgermacrene A (**1**) in $CDCl_3$ (600 MHz).

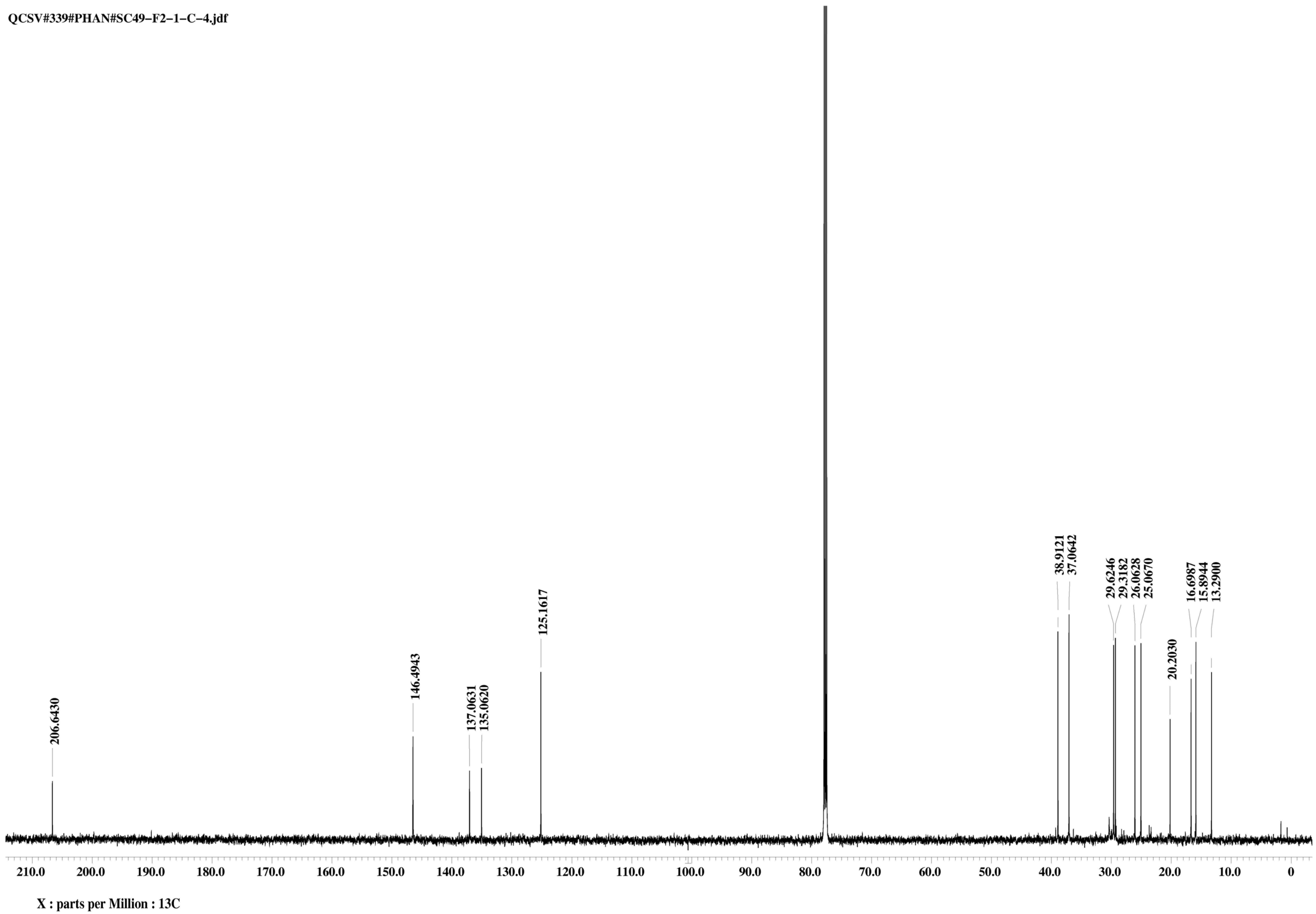

Figure S2. ^{13}C-NMR spectrum of capgermacrene A (**1**) in $CDCl_3$ (150 MHz).

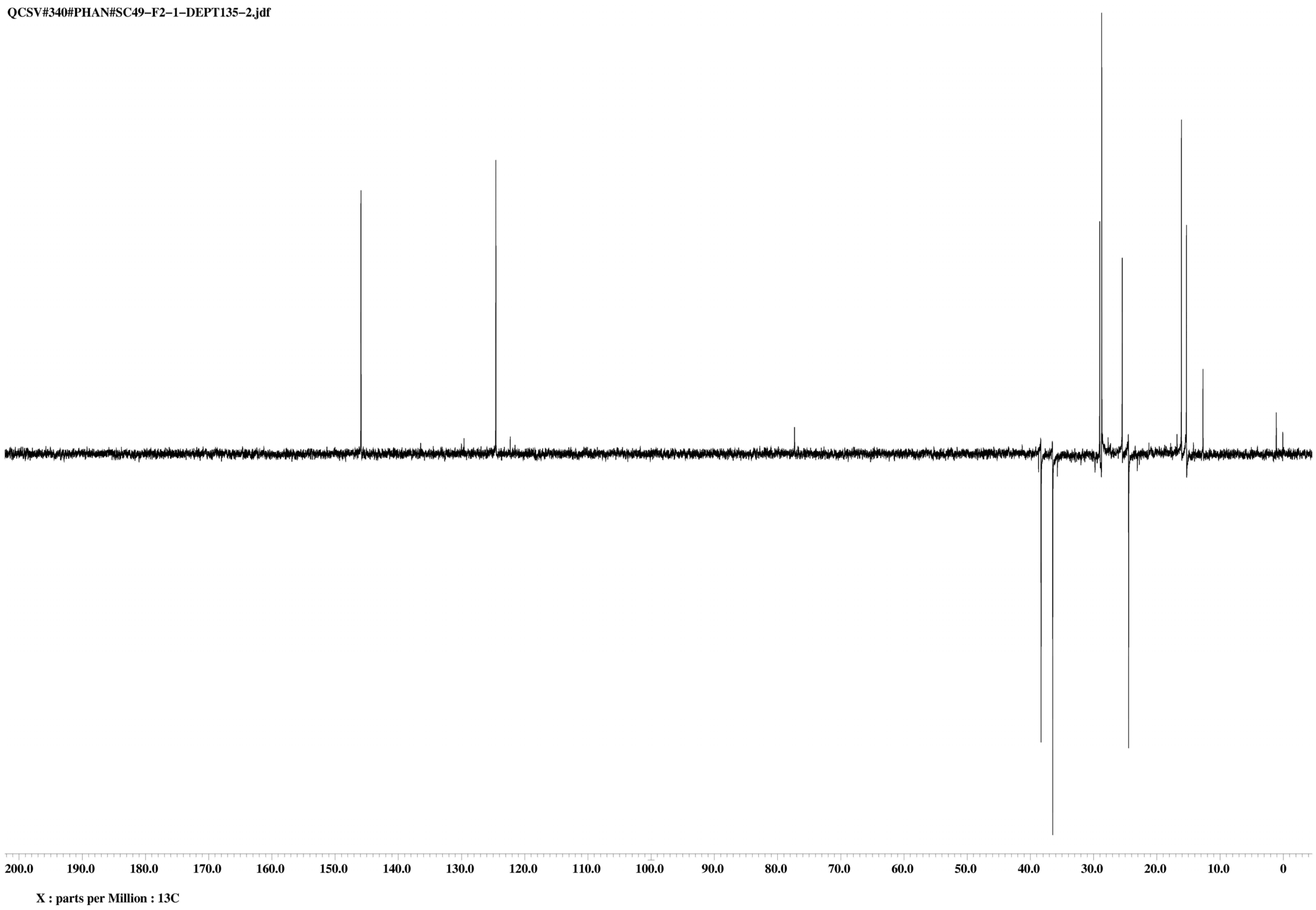

Figure S3. DEPT-135 spectrum of capgermacrene A (**1**) in $CDCl_3$.

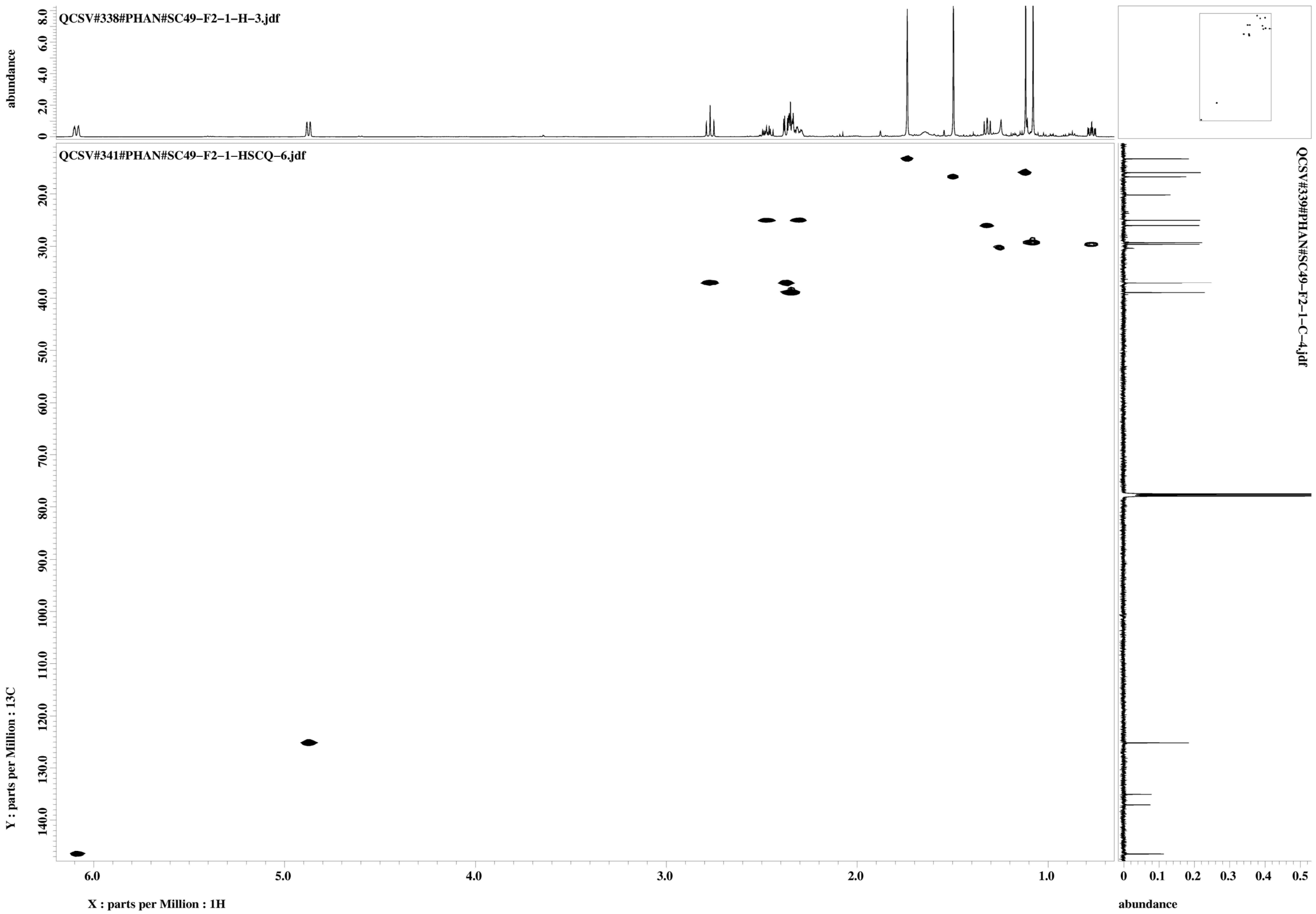

Figure S4. HSQC spectrum of capgermacrene A (**1**) in $CDCl_3$.

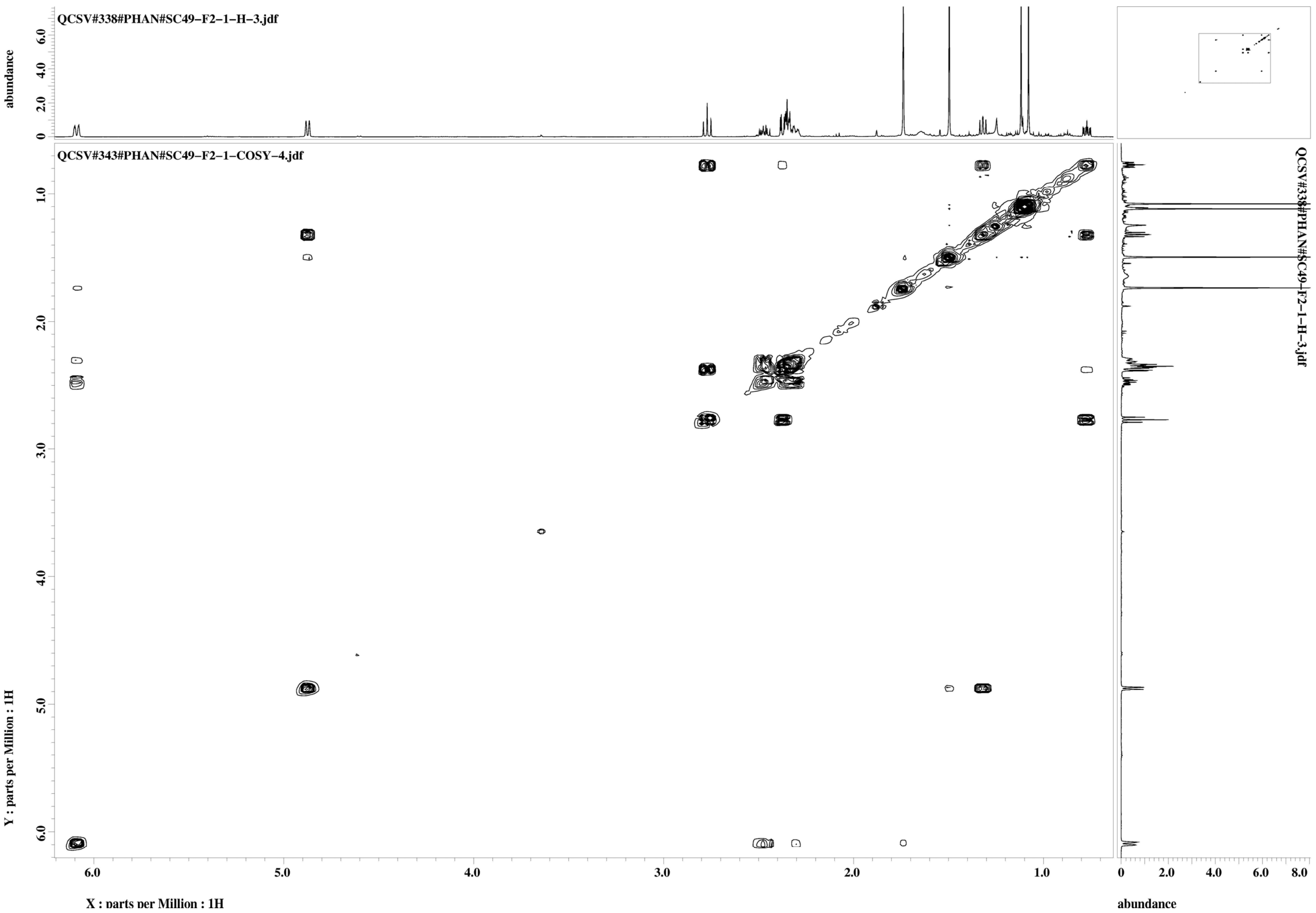

Figure S5. ^{1}H-^{1}H COSY spectrum of capgermacrene A (**1**) in $CDCl_3$.

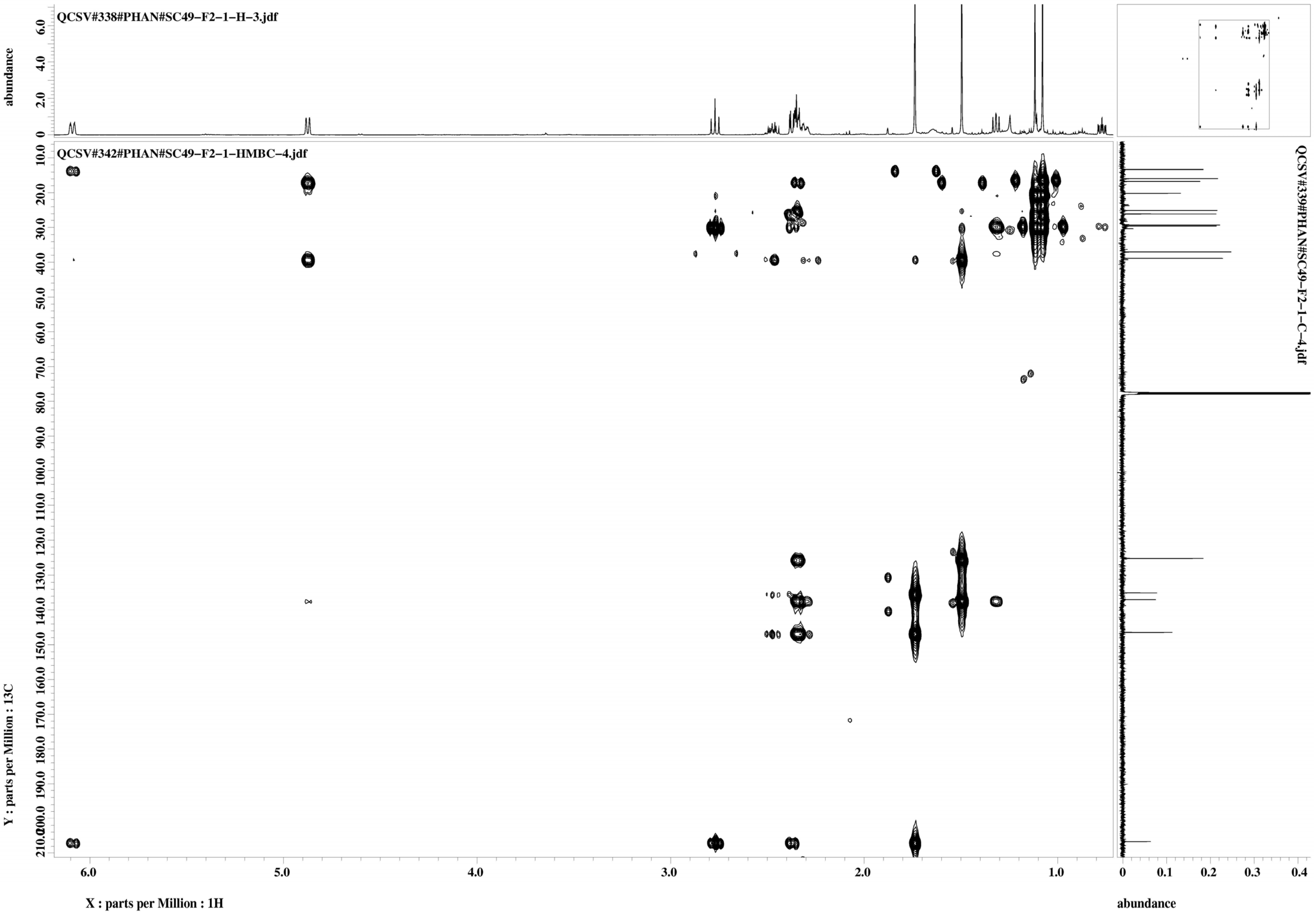

Figure S6. HMBC spectrum of capgermacrene A (**1**) in $CDCl_3$.

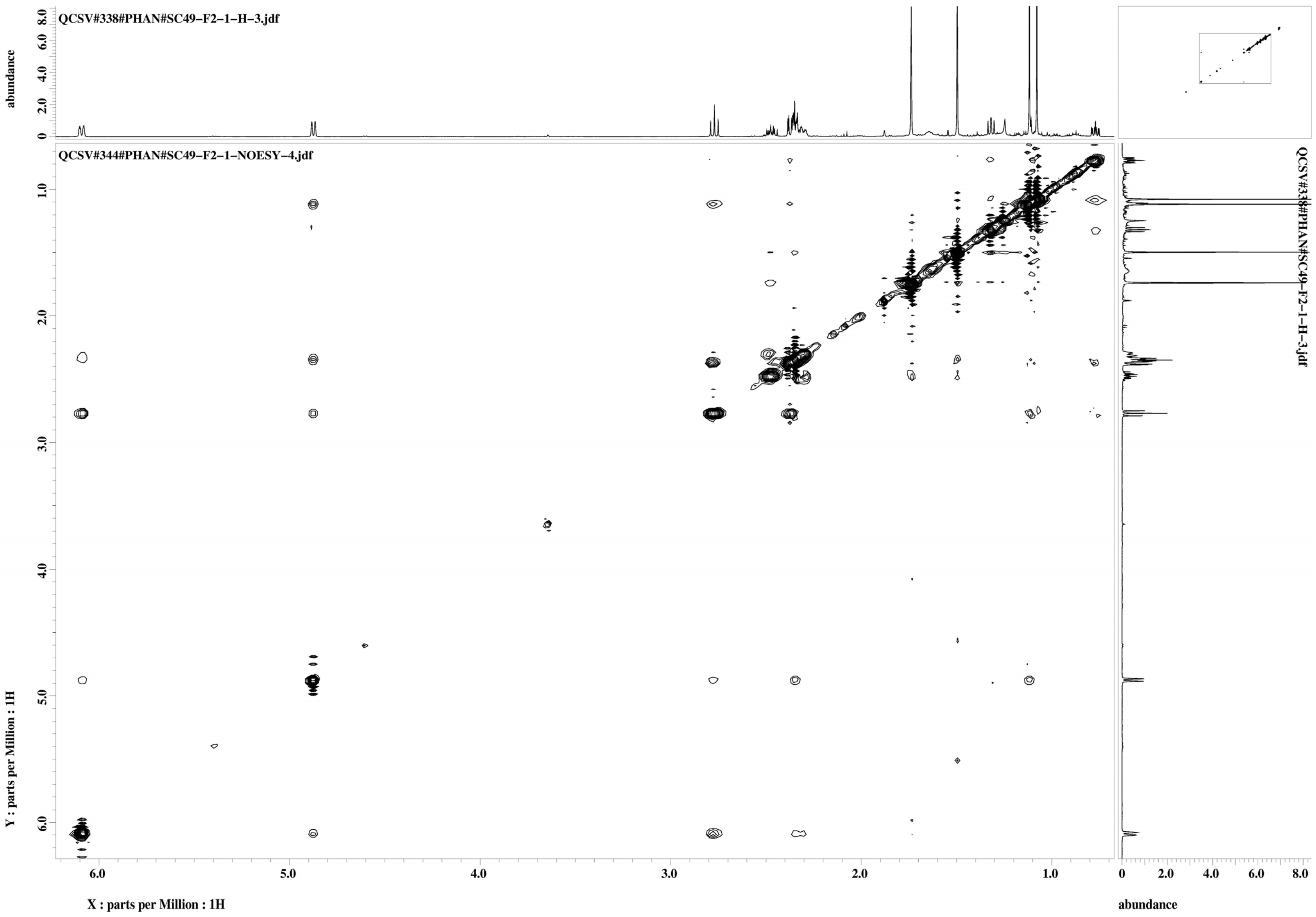

Figure S7. NOESY spectrum of capgermacrene A (**1**) in $CDCl_3$.

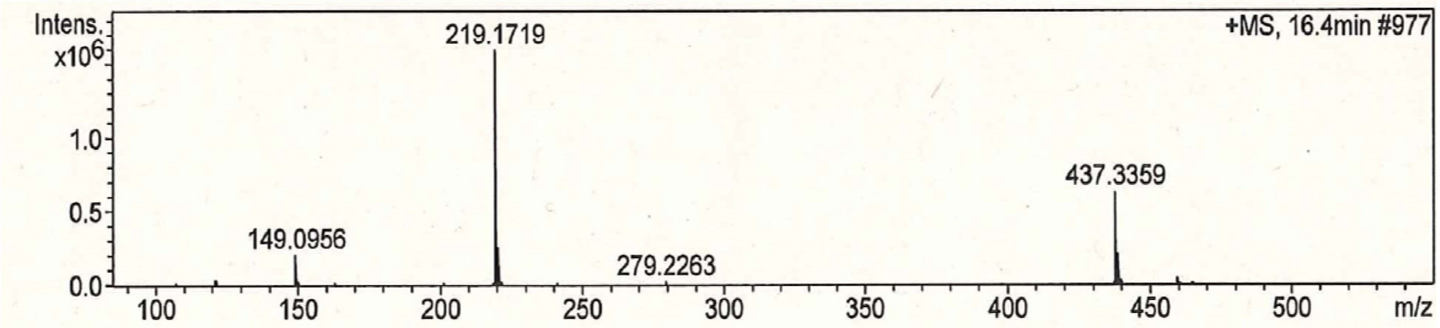

Meas. m/z	#	Formula	Score	m/z	err [mDa]	err [ppm]	mSigma	rdb	e⁻ Conf	N-Rule
219.1719	1	C 15 H 23 O	91.61	219.1743	2.4	11.0	2.5	4.5	even	ok
	2	C 10 H 23 N 2 O 3	100.00	219.1703	-1.6	-7.3	28.8	0.5	even	ok
	3	C 9 H 23 N 4 O 2	0.00	219.1816	9.6	43.9	30.9	0.5	even	ok

Figure S8. HR-TOFMS data of capgermacrene A (**1**).

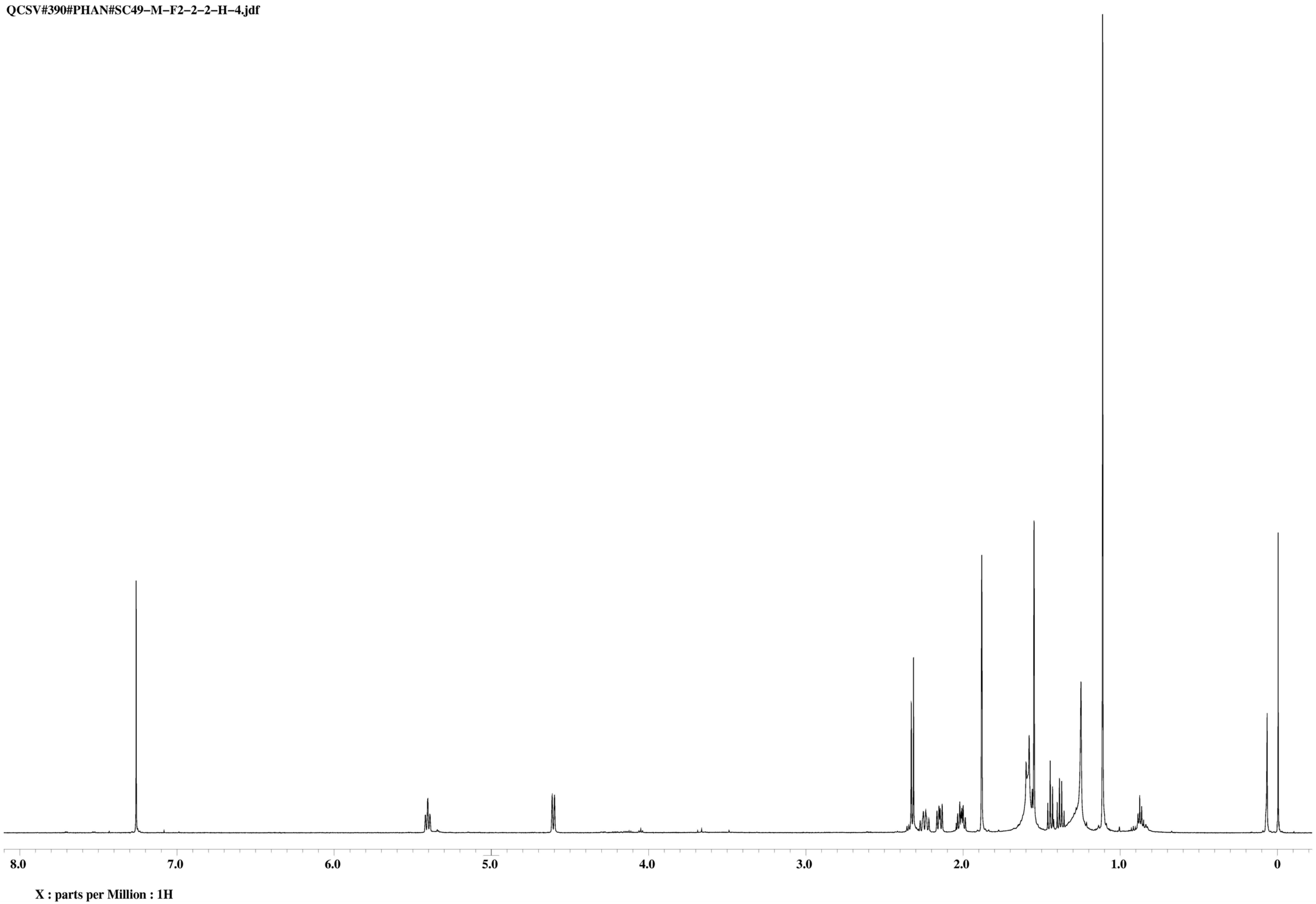

Figure S9. ^{1}H-NMR spectrum of capgermacrene B (**2**) in $CDCl_3$ (600 MHz).

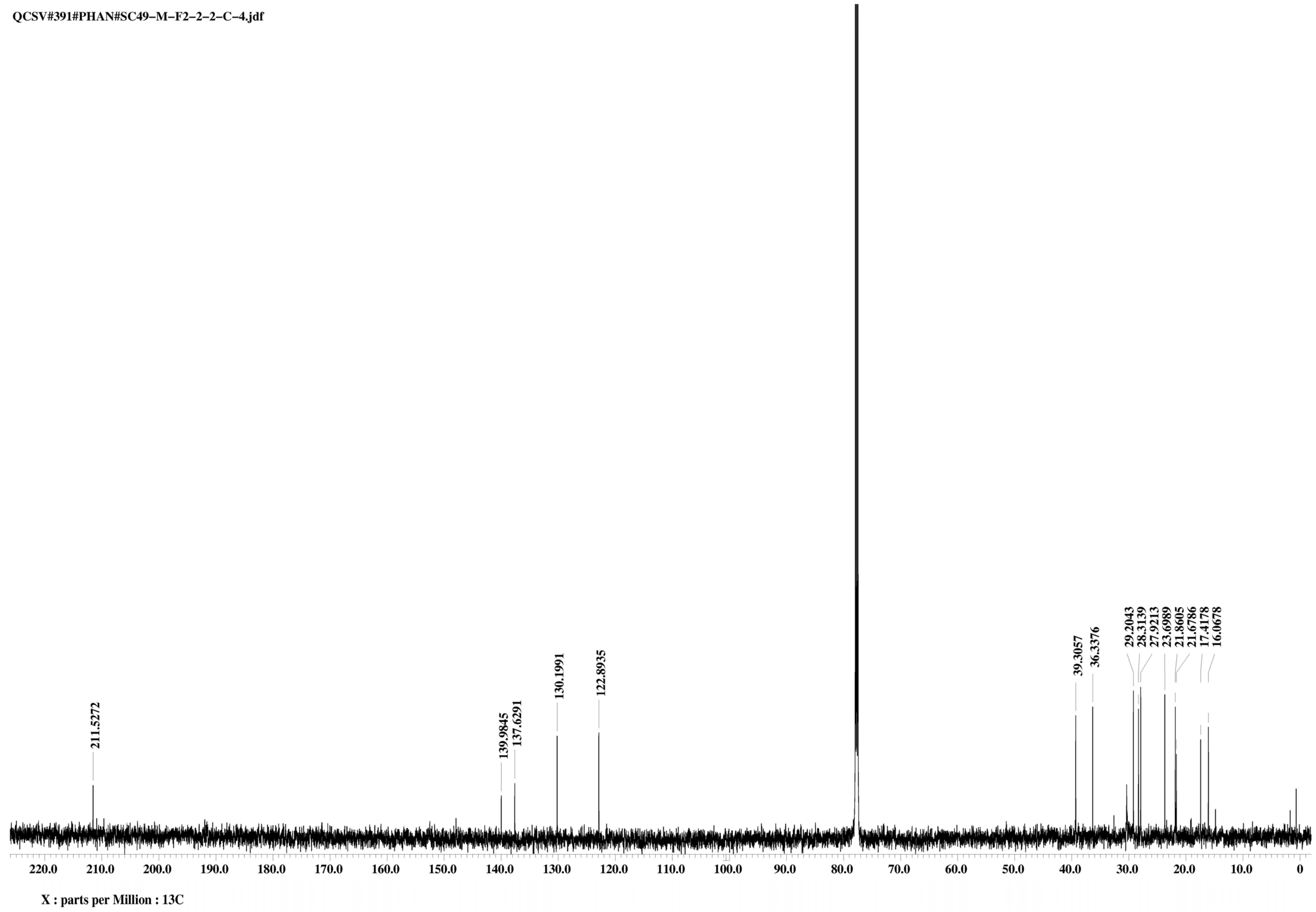

Figure S10. ^{13}C-NMR spectrum of capgermacrene B (**2**) in $CDCl_3$ (150 MHz).

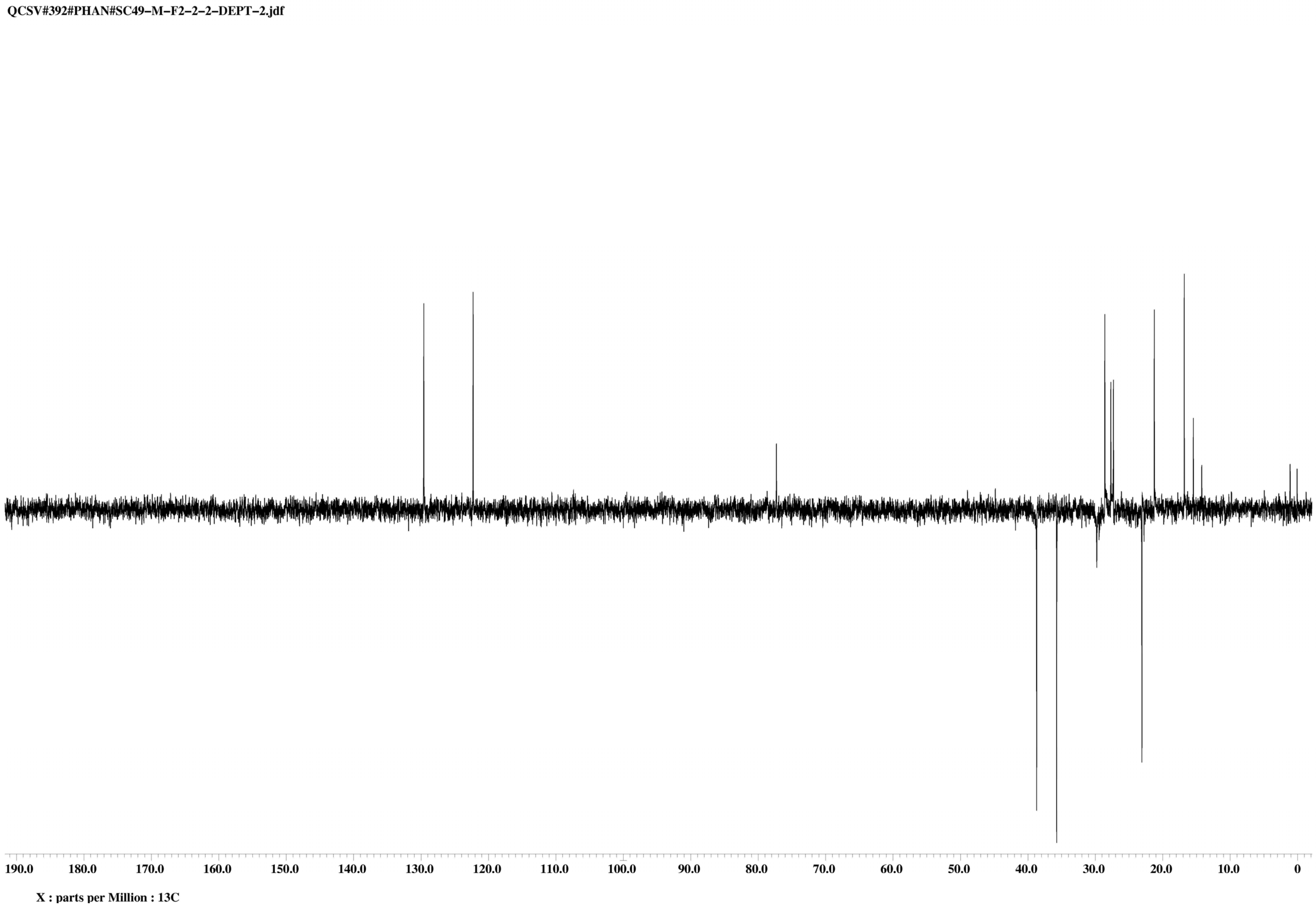

Figure S11. DEPT-135 spectrum of capgermacrene B (**2**) in $CDCl_3$.

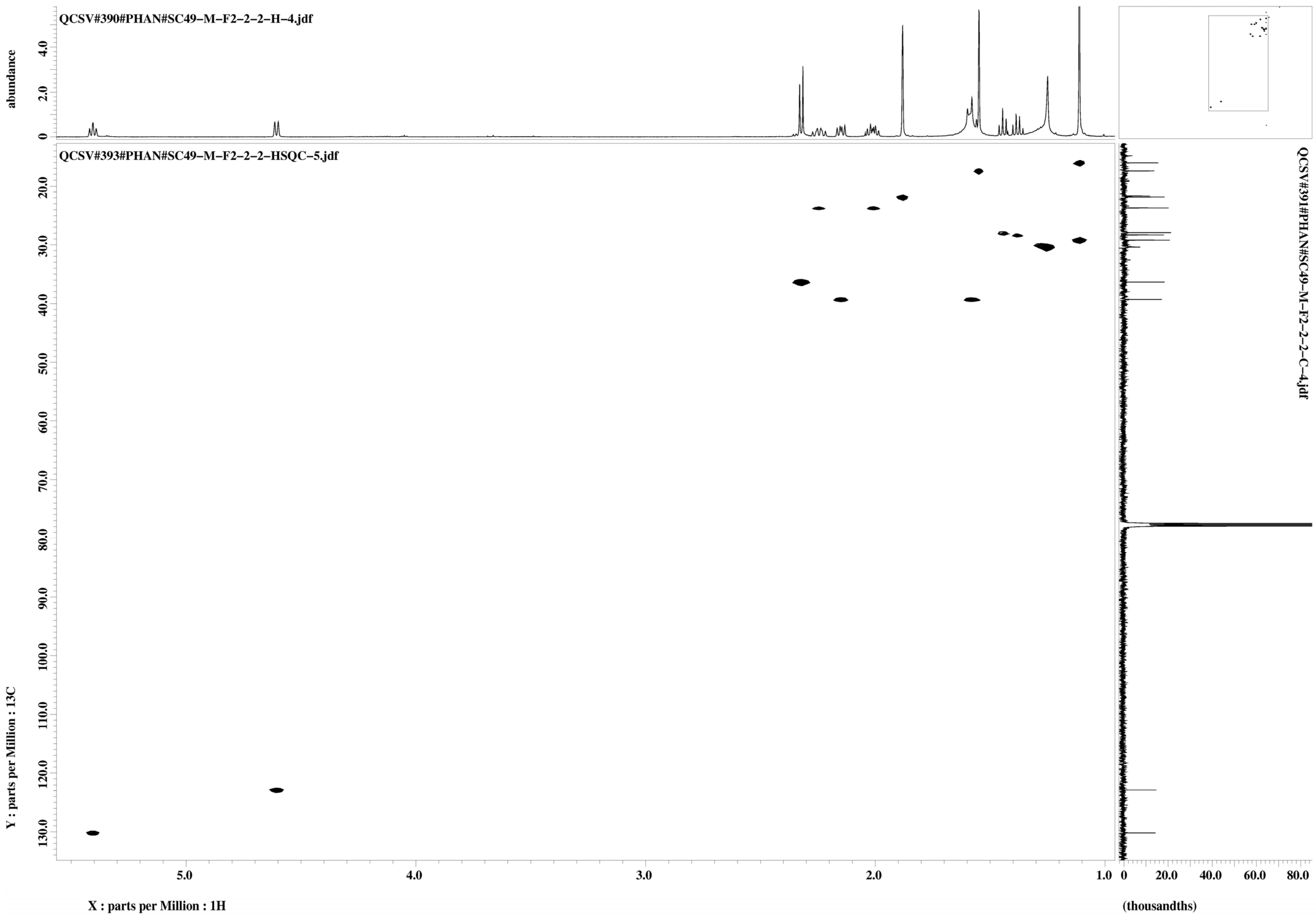

Figure S12. HSQC spectrum of capgermacrene B (**2**) in $CDCl_3$.

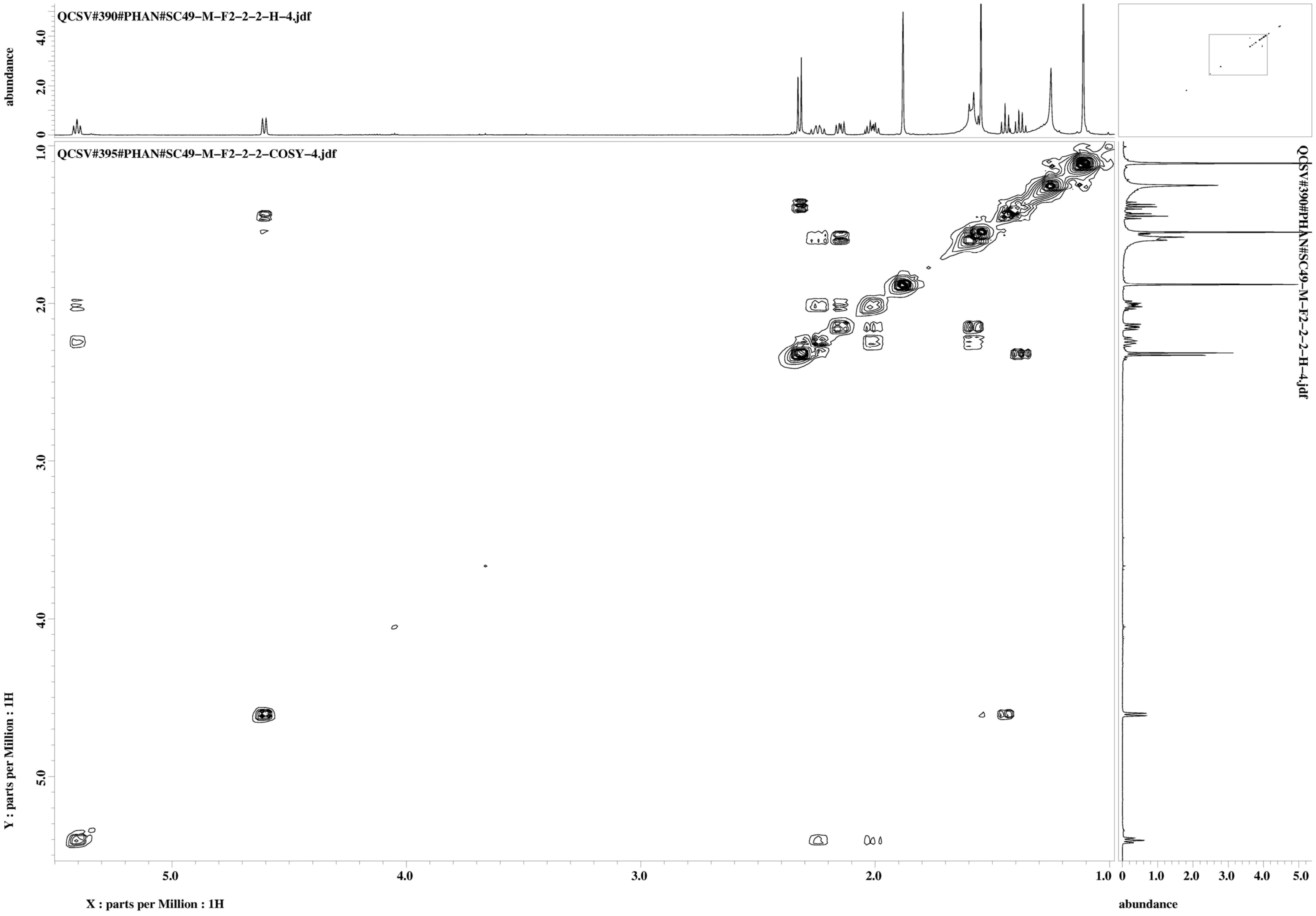

Figure S13. ^{1}H-^{1}H COSY spectrum of capgermacrene B (**2**) in $CDCl_3$.

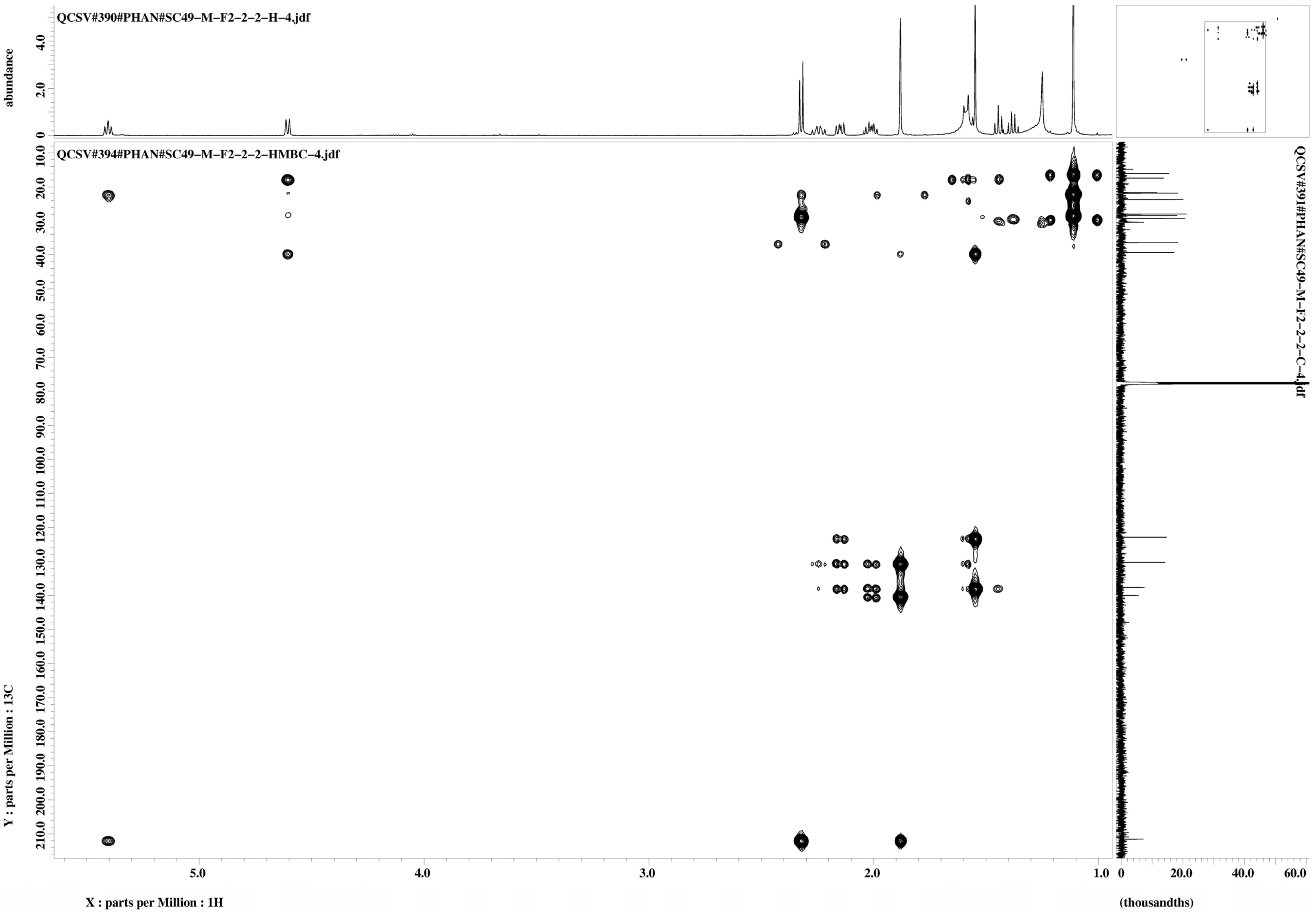

Figure S14. HMBC spectrum of capgermacrene B (**2**) in $CDCl_3$.

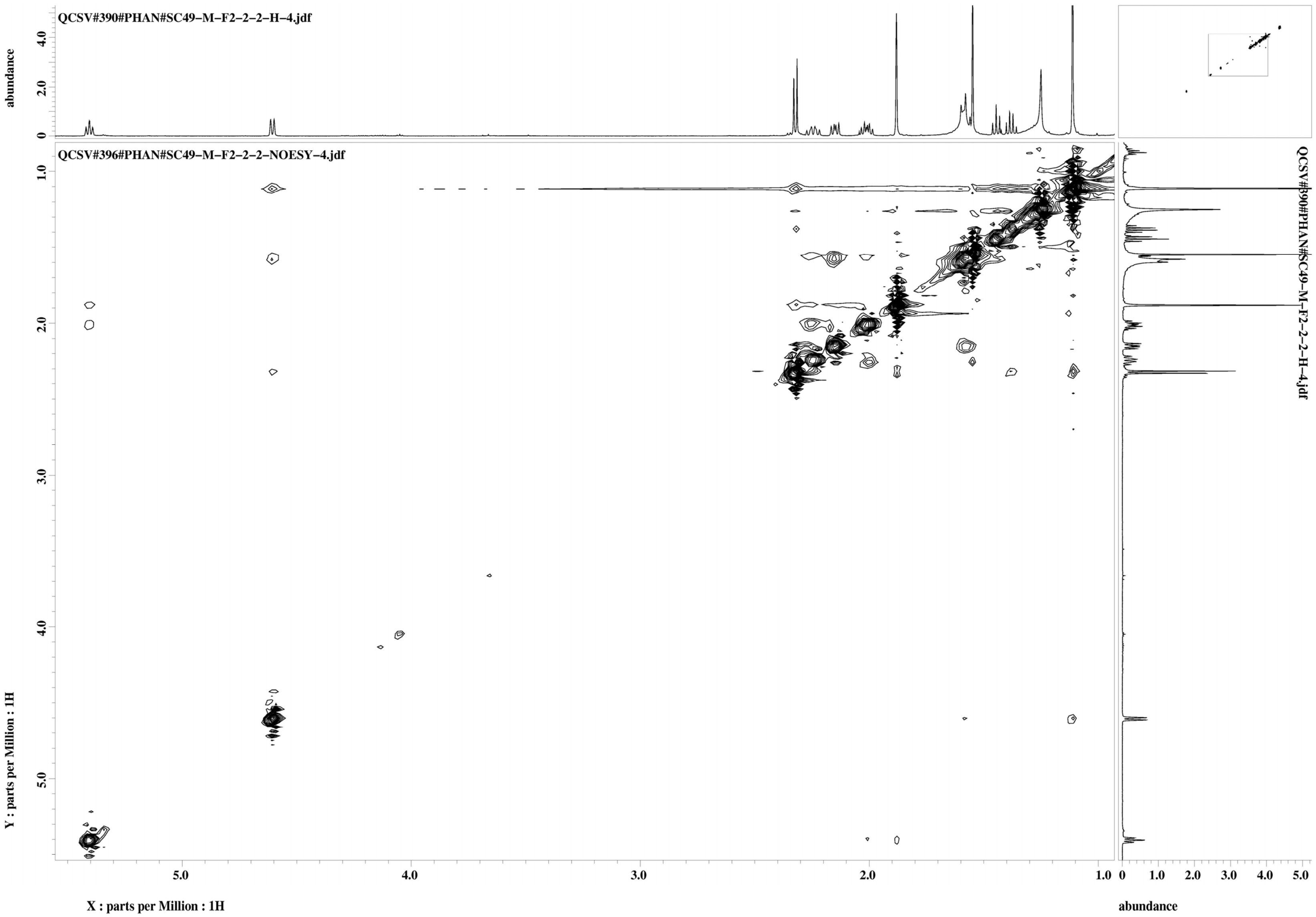

Figure S15. NOESY spectrum of capgermacrene B (**2**) in $CDCl_3$.

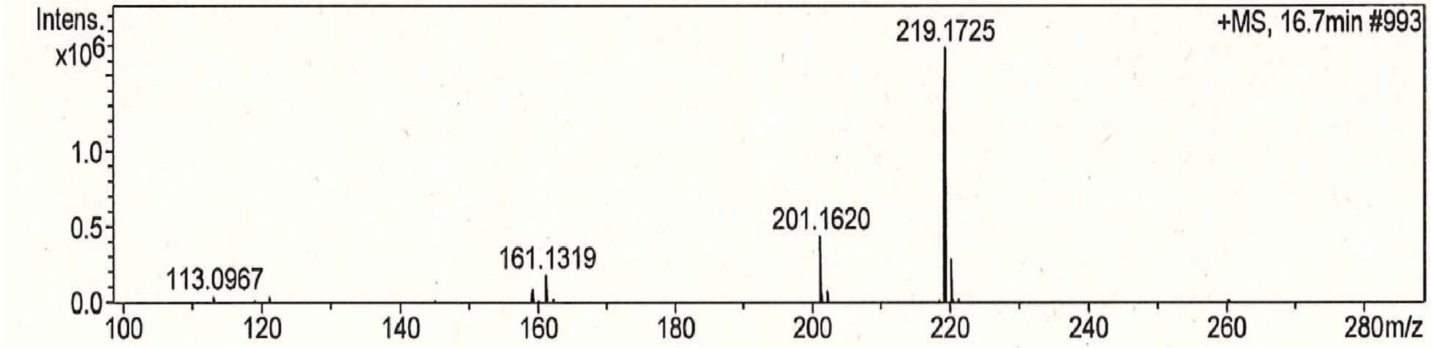

Meas. m/z	#	Formula	Score	m/z	err [mDa]	err [ppm]	mSigma	rdb	e⁻ Conf	N-Rule
201.1620	1	C 15 H 21	100.00	201.1638	1.8	9.0	1.7	5.5	even	ok
	2	C 10 H 21 N 2 O 2	43.71	201.1598	-2.2	-11.0	27.4	1.5	even	ok
	3	C 9 H 21 N 4 O	0.00	201.1710	9.0	44.8	29.7	1.5	even	ok
219.1725	1	C 15 H 23 O	100.00	219.1743	1.9	8.6	4.8	4.5	even	ok
	2	C 10 H 23 N 2 O 3	47.69	219.1703	-2.1	-9.7	31.5	0.5	even	ok

Figure S16. HR-TOFMS data of capgermacrene B (**2**).

© 2015 by the authors; licensee MDPI, Basel, Switzerland. This article is an open access article distributed under the terms and conditions of the Creative Commons Attribution license (http://creativecommons.org/licenses/by/4.0/).
